# Protein Hydrolyzates from Changbai Mountain Walnut (*Juglans mandshurica* Maxim.) Boost Mouse Immune System and Exhibit Immunoregulatory Activities

**DOI:** 10.1155/2018/4576561

**Published:** 2018-05-28

**Authors:** Jing Li, Ji Wang, Chunlei Liu, Li Fang, Weihong Min

**Affiliations:** ^1^College of Food Science and Engineering, Jilin Agricultural University, Changchun, Jilin Province 130118, China; ^2^National Engineering Laboratory of Wheat and Corn Deep Processing, Changchun, Jilin Province 130118, China; ^3^Sports Health Technology College, Jilin Sports University, Jilin, China

## Abstract

The Changbai Mountain walnut (*Juglans mandshurica *Maxim.) is a rich source of essential amino acids. Walnut dregs are byproducts of edible oil production and primarily used as fodder and fertilizers. We systematically examined the effect of three types of walnut protein hydrolyzates—albumin, glutelin, and globin—on the immune system of mice and aimed to provide the theoretical basis for developing and utilizing* J. mandshurica *Maxim. protein resources. In comparison with the normal control mice, those treated with different doses of walnut proteins showed improved immune indices, including organ index, spleen lymphocyte proliferation, macrophage activity, number of CD4^+^ and CD8^+^ T cells, immunoglobulin A (IgA) and secretory IgA content, and mRNA and protein expression levels of cytokine factors. Our results indicated that these walnut proteins may have positive effects on the immune system and perform their immunomodulatory functions by inducing splenic enlargement. These findings support the use of walnut proteins as nutritional sources to boost the immune system.

## 1. Introduction

Proteins are large macromolecules with specific three-dimensional structures, which not only constitute the body composition and metabolism basis but also served as important components of the human diet [1]. Most proteins in human diet are derived from animal sources. In recent years, proteins from plants have attracted attention owing to their specific physiological functions, balanced amino acid composition, high content of essential amino acids, no cholesterol, and lower price as compared with those from animals [2]. Walnut is a nutrient-rich food that is widely planted in China. The Changbai Mountain walnut (*Juglans mandshurica *Maxim) belongs to the Chinese walnut catalpa walnut genera of the deciduous broad-leaf tree species. The protein content of this species is usually 14%–17% (maximum of 29.7%), while its digestion rate is 85% and above [3]. At least 18 types of proteins have been identified from walnut till now and eight of these are rich in essential amino acids, including arginine and glutamic acid. The content of albumin, prolamin, globin, and glutelin in total walnut proteins is 6.81%, 5.33%, 17.57%, and 70.11%, respectively [3].

Immunomodulation has gathered increasing attention in recent years, as the immune response plays a vital role in preventing diseases. The concept of immunotherapy was conceived over a century ago, and significant progress has been made in unraveling it in recent years [4]. The host defense response may be enhanced by immunomodulators, resulting in the enhancement of disease resistance [5]. Bioactive peptides of short segments (usually 3–20 amino acids) that are derived from plants and animals have been recently shown to exhibit immune-boosting immunomodulatory properties. These peptides may positively influence human health [6]. To date, various bioactive peptides prepared using protease-mediated hydrolysis have been identified, including angiotensin-converting enzyme inhibitory peptides, antioxidant peptides, mineral-binding peptides, immunomodulatory peptides, antithrombotic peptides, and antimicrobial peptides [7, 8]. Of these, immunomodulatory peptides are known to stimulate the proliferation of lymphocytes, increase the phagocytic activity of macrophages, and enhance the immunity of several organisms [9].

Walnut dregs (containing around 58% protein content) are byproducts of edible oil production and primarily used as fodder and fertilizers. These dregs are considered to have low value but are high-quality protein resources. We systematically examined the effect of three types of walnut protein hydrolyzates—albumin, glutelin, and globin—on the immune system of mice and aimed to provide the theoretical basis for developing and utilizing* J. mandshurica *Maxim. protein resources and experimental basis for developing new plant protein sources.

## 2. Materials and Methods

### 2.1. Materials and Reagents

Walnut dregs were prepared in our laboratory. Folin-Phenol Protein Quantitative Kit was obtained from Beijing Dingguo Biological Engineering Co. (Beijing, China) and alcalase from Denmark Novozymes (Copenhagen, Denmark). Hank's liquid was procured from Genview (Tallahassee, FL, USA), while Roswell Park Memorial Institute -(RPMI-) 1640 culture medium was purchased from HyClone (Logan, UT, USA). Dimethyl sulfoxide (DMSO), methylthiazolyldiphenyl-tetrazolium bromide (MTT), and concanavalin A were purchased from Sigma (St. Louis, MO, USA) and fetal bovine serum (FBS) was acquired from Gibco (Grand Island, NY, USA). The antibody used for western blot analysis was provided by BD Bioscience (San Diego, CA, USA). All other reagents used were of analytical grade.

### 2.2. Preparation of Albumin, Glutelin, and Globin

The three proteins were isolated by the Osborne method [10] using walnut protein isolate (protein content of 74%) as the raw material. Distilled water was added to walnut protein isolate at a ratio of 10 : 1 (v/w) and the extract was magnetically stirred for 1 h. After being centrifuged at 5,000 rpm for 10 min, the supernatant was extracted. The remaining precipitate was magnetically stirred for another 1 h with distilled water at a ratio of 1 : 10 (w/v), and the supernatant was reextracted. The extract was mixed and its isoelectric point was adjusted to 4.1, followed by its incubation for 30 min. The extract was washed thrice to adjust its pH to neutral and freeze-dried as albumin.

After albumin extraction, the remaining precipitate was extracted thrice with 1 mol/L sodium chloride (NaCl); the three extracts were combined and the isoelectric point of the resulting suspension was adjusted to 4.3 after incubation for 30 min. The pH of the extract was adjusted to neutral after washing thrice. The extract was placed into a dialysis bag and distilled water was changed after every 3 h. The solution was dialyzed at 4°C for 3 days to remove salt ions from proteins. After freeze drying, globin was obtained.

To obtain glutelin, the pH of the remaining precipitate was adjusted thrice with 1% sodium hydroxide (NaOH). The supernatant was centrifuged and the pH was adjusted to 4.6 by incubating for 30 min, followed by centrifugation at 5,000 rpm for 10 min. The extract was washed thrice to adjust the pH to 7.0 and freeze-dried to obtain glutelin.

### 2.3. Sodium Dodecyl Sulfate- (SDS-) Polyacrylamide Gel Electrophoresis (PAGE)

We performed SDS-PAGE as previously described [11]. Briefly, three protein samples (1 mg/mL, 20 *μ*L) were placed on biphasic polyacrylamide gels (15%). The electrode buffer was Tris-HCl (pH 8.3) and protein molecular weight marker containing ten proteins was used as the standard at a volume of 10 *μ*L. Runs were performed at voltages of 120 and 60 V for separating gel and stacking gel, respectively. Coomassie Brilliant Blue R-250 was used for gel staining and Molecular Imager FX (Bio-Rad, USA) was used for image capturing.

### 2.4. Animal Care and Experimental Design

For animal study, 110 healthy adult BALB/c female mice weighing 18–20 g were obtained from Changchun Institute of Biological Products Co., Ltd. (License No. SCXK [Liao] 2015–0001, Changchun, China). Mouse feed was purchased from Changchun YiSi Experimental Animal Technology Co., Ltd. (License No. SCXK [Ji] 2016–0005, Changchun, China). All animal experiments were performed as per the Guide for the Care and Use of Laboratory Animals and Animal Welfare Act. Mice were randomly placed in polypropylene cages (320 × 215 × 170 mm) with stainless steel covers and housed under 22 ± 2°C and a relative humidity of 50 ± 10% and 12 h light/dark cycle, with lights off at 8 pm. Mice were fed with a standard diet and running water was supplied ad libitum.

After acclimatization for 1 week, mice were randomly assigned to 11 groups (*n* = 10 in each group). Mice from the experimental groups were fed with different doses of glutelin, albumin, and globin once a day at 200 (low dose, L), 400 (moderate dose, M), and 800 (high dose, H) mg/kg body weight (bw) (Glu + H, Glu + M, Glu + L, Alb + H, Alb + M, Alb + L, Glo + H, Glo + M, and Glo + L) for 35 days. In the positive control group, thymopeptide was administered at a dose of 0.30 mg/mL/kg bw. In the normal control group, mice were administered with 0.2 mL sterile saline. Food intake was recorded once a day, and body weight was measured thrice a week during the experiment.

### 2.5. Tissue Collection and Analysis

After 35 days of feeding, mice were starved for 12 h, weighed, and subjected to blood collection. After blood collection, mice were immediately killed with cervical dislocation. RPMI-1640 with 10% heat-inactivated FBS (3 mL) was injected into the mice; the peritoneal cavity was infused with cold phosphate-buffered saline (PBS; 10 mL) and the abdomen was gently massaged for 2 min. Peritoneal macrophages were collected. Visceral organs, including the spleen and thymus, were aseptically harvested and weighed. Absolute and relative weights of the visceral organs from each experimental group were calculated. Splenocyte cultures were prepared from the spleen samples for further analysis.

### 2.6. Thymus and Spleen Indices

Thymus and spleen indices were determined as previously described [12]. Briefly, immune organs, including the spleen and thymus, were collected and weighed immediately after the mice were sacrificed. The following formula was used for calculating thymus and spleen indices: index (mg/g) = (weight of the thymus/weight of the spleen)/bw.

### 2.7. Determination of Phagocytosis by Peritoneal Macrophages

Phagocytosis by peritoneal macrophages was determined according to a previous study [13]. Briefly, peritoneal macrophages were washed twice with PBS and 1 × 10^6^ cells/mL were incubated for 2 h at 37°C with 5% CO_2_ in a humidified incubator in 96-well plates to purify macrophages. To eliminate nonadherent cells, RPMI-1640 was used to wash purified cells. Each well was treated with 100 *μ*L of lysis buffer (acetic acid and alcohol; 1 : 1, v/v) and the samples were overnight incubated at 4°C. The absorbance of each well was measured at 540 nm wavelength to evaluate the phagocytic index.

### 2.8. Lymphocyte Proliferation Assay

Spleen tissues were aseptically excised from the mice and single-cell suspensions were obtained after gently pressing the sample with a syringe core and passing it through a 200-mesh steel sieve. Red blood cell lysis buffer was used to remove erythrocytes and PBS was used to wash splenocytes thrice. The suspensions were centrifuged at 1,000 rpm for 5 min. RPMI-1640 medium containing 10% newborn bovine serum was added to splenocytes and cells were seeded at a density of 1 × 10^6^ cells/mL in 96-well plates with concanavalin A (5 *μ*g/mL) and incubated for 72 h at 37°C under 5% CO_2_ in a humidified incubator. Before the termination of cell culture, 10 *μ*L of 5 mg/mL MTT was added to each well and samples were continuously incubated for 4 h. After removing the supernatant, 150 *μ*L of DMSO was added to each well and the absorbance was measured at 570 nm wavelength within 10 min using a microplate reader. Splenocyte proliferation was evaluated as follows: stimulation index = OD_570_ of stimulated cells/OD_570_ of the negative control [14].

### 2.9. Determination of CD4^+^ and CD8^+^ T-Cell Subtypes

The determination of CD4^+^ and CD8^+^ T lymphocytes was performed with flow cytometry following the study of Ren et al. [15].

### 2.10. Measurement of Intestinal Secretory Immunoglobulin A (sIgA) Levels

Intestinal samples, including 10 cm of the caecum and distal sections, were collected after mice euthanization. These samples were homogenized in 1 mL of PBS supplemented with 0.1% bovine serum albumin. The particulate material in the solution was removed by centrifugation for 15 min at 4°C and 1,410 ×g. The supernatant was collected and stored at −20°C for measuring sIgA levels with an enzyme-linked immunosorbent assay (ELISA).

### 2.11. Relative mRNA Expression Levels of Interferon- (IFN-) *γ* and Interleukin- (IL-) 6 in the Spleen

We performed quantitative reverse transcription polymerase chain reaction (qRT-PCR) as previously described [16]. Total RNA from the mouse spleen was extracted with TRIzol (Invitrogen, Carlsbad, CA). Real-time qPCR was performed using a real-time rotary analyzer using the following program: 15 min at 95°C for hot-start activation, followed by 45–50 cycles of 30 s at 95°C for denaturation, 30 s at 60°C for annealing, and 30 s at 72°C for extension. Primers specific for IL-6 and IFN-*γ* are shown in Table 1. The reactions were performed in triplicate, and results of three independent experiments are shown.

### 2.12. Serum Levels of IL-6, IFN-*γ*, and IgA

Blood samples from the eyes of mice were coagulated at 25°C. The serum was separated by centrifugation for testing. The concentrations of serum cytokines, including IL-6, IFN-*γ*, and IgA, were determined using ELISA kits (R&D, USA).

### 2.13. Statistical Analysis

All experiments were performed in triplicate. Data analysis was conducted using SPSS (SPSS, Version 11.5, SPSS Inc., Chicago, IL). Multiple comparisons among groups were conducted with one-way analysis of variance (ANOVA) followed by Tukey-Kramer post hoc test. Values are shown as mean ± standard deviation (SD). A value of *P* < 0.05 was regarded as statistically significant.

## 3. Results

### 3.1. Sequential Extraction and Electrophoresis of Proteins

**Figure 1:**
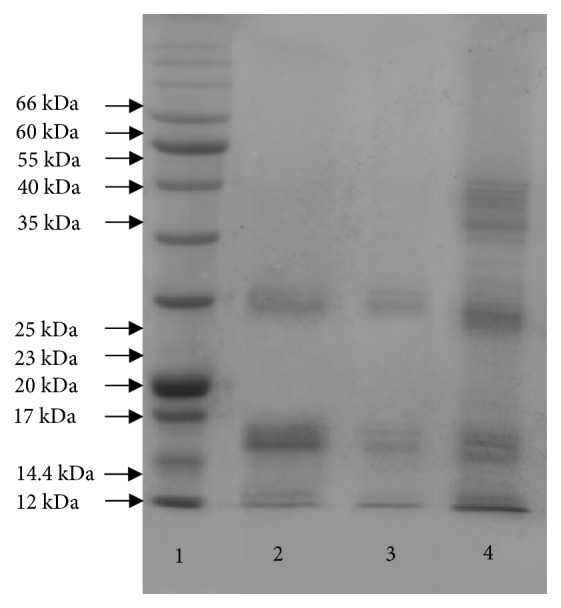
Electrophoretic banding protein generated by SDS-PAGE of walnut proteins. 1, 2, 3, and 4 swim lanes correspond to standard proteins, albumin, glutelin, and globin.

Three proteins were extracted and represented with some accessions illustrated in Figure 1. The molecular weights of albumin and glutelin ranged from 11 to 35 kDa, while globin had molecular weight between 11 and 63 kDa. These results indicate the successful extraction of the three proteins.

### 3.2. Effect of Walnut Proteins on Immune Organs of Mice

Thymus and spleen are important immune organs and their indices may reflect the immune function of a particular organism. The effects of walnut proteins on the spleen index of mice are shown in Table 2. In comparison with the control group, high-dose globin group showed a significant increase in the spleen index (*P* < 0.05). Spleen index was significantly increased in all albumin groups (*P* < 0.01). The spleen index in the moderate-dose albumin group was close to that in the positive control group. In addition, the spleen index was remarkably affected in all glutelin groups, with a significant level observed for the high-dose glutelin group (*P* < 0.01).

Thymus index of mice administered with walnut proteins is shown in Table 3. The results indicate that moderate- and high-dose globin treatment resulted in a significant increase in the thymus index (*P* < 0.05). Albumin had a remarkable effect on the thymus index at all doses (*P* < 0.05); thymus index in the moderate-dose group was highly significant (*P* < 0.01) and increased up to 82.5% as compared with the control group and 4.4% as compared with the positive control group. Glutelin also significantly increased the thymus index at all doses (*P* < 0.05). These findings indicate that walnut globin, albumin, and glutelin significantly affected the immune organs.

### 3.3. Phagocytic Activity of Macrophages

**Figure 2:**
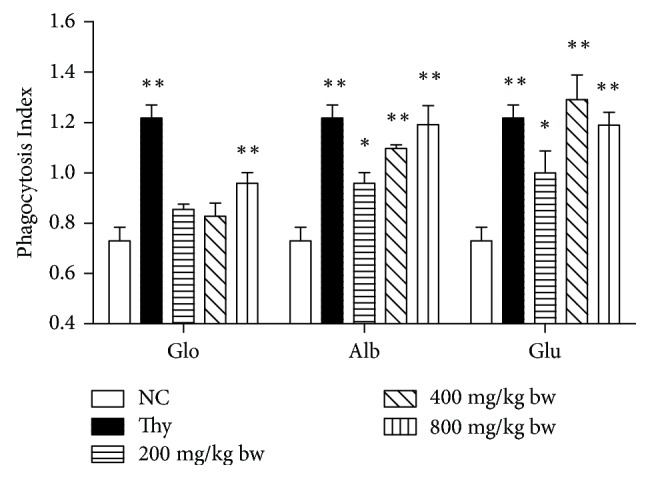
Effects of walnut proteins on pinocytosis of peritoneal macrophages. Peritoneal macrophages were prepared from mice treated with walnut proteins at different doses. A distilled water-treated group was used as the negative control and thymopeptide was used to treat the positive control group. The phagocytosis index was measured using the accumulation of neutral red in cells via a colorimetric assay, which represented the phagocytic activity of the peritoneal macrophages of each protein-treated group. Values are expressed as mean ± standard deviation (SD). Asterisks represent statistically significant differences; ^*∗*^*P* < 0.05 and ^*∗∗*^*P* < 0.01 compared with the control group.

The effects of walnut proteins on the phagocytic activity of macrophages were determined (Figure 2). In comparison with the control group, high-dose globin group showed a significant increase in the phagocytic activity of macrophages (*P* < 0.01). All albumin and glutelin groups showed significantly increased phagocytic activity of macrophages. Furthermore, the phagocytic activity reported in the high-dose albumin and moderate- and high-dose glutelin group was close to that observed for the positive control group (*P* > 0.05). In particular, the phagocytosis index in the moderate-dose glutelin group was significantly higher than that in the positive control group. Thus, walnut proteins may increase the macrophage activity and promote resistance to infection by enhancing the innate immune response.

### 3.4. Lymphocyte Proliferation

**Figure 3:**
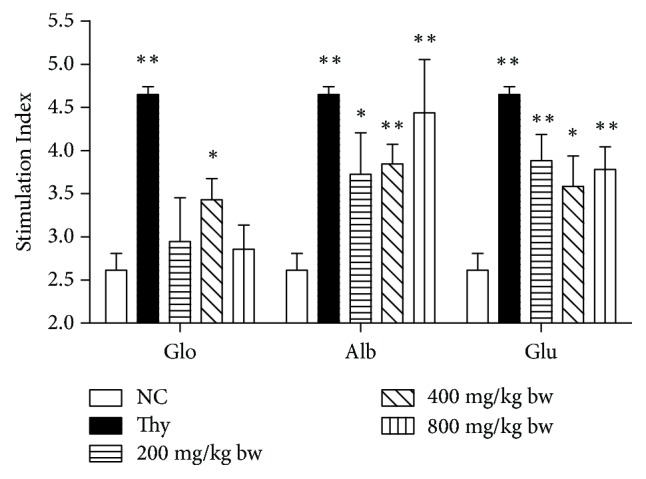
Proliferative analysis of splenocytes from mice treated with the walnut proteins at different doses. Approximately 5 × 10^5^ splenocytes were isolated from the protein-treated mice. A distilled water-treated group was used as the negative control, and thymopeptide was used as the positive control. The stimulation index represents the proliferative capacity of splenocytes from each group after treatment with the walnut proteins. Values are expressed as mean ± SD. Asterisks represent statistically significant differences; ^*∗*^*P* < 0.05 and ^*∗∗*^*P* < 0.01 compared with the control group.

We evaluated the lymphocyte proliferation rate in different groups (Figure 3). In comparison with the control group, the moderate-dose globin group clearly influenced the spleen lymphocyte proliferation (*P* < 0.05). No significant effect on spleen lymphocyte proliferation was observed in the low- and high-dose globin groups (*P* > 0.05). In addition, all doses of albumin and glutelin significantly increased spleen lymphocyte proliferation (*P* < 0.05). In comparison with the control treatment, high-dose albumin treatment significantly increased the lymphocyte proliferation by up to 50.4% (*P* < 0.05), a value close to that reported for the positive control group.

### 3.5. Effect of Walnut Proteins on Mouse CD4^+^ and CD8^+^ T Cells

**Figure 4:**
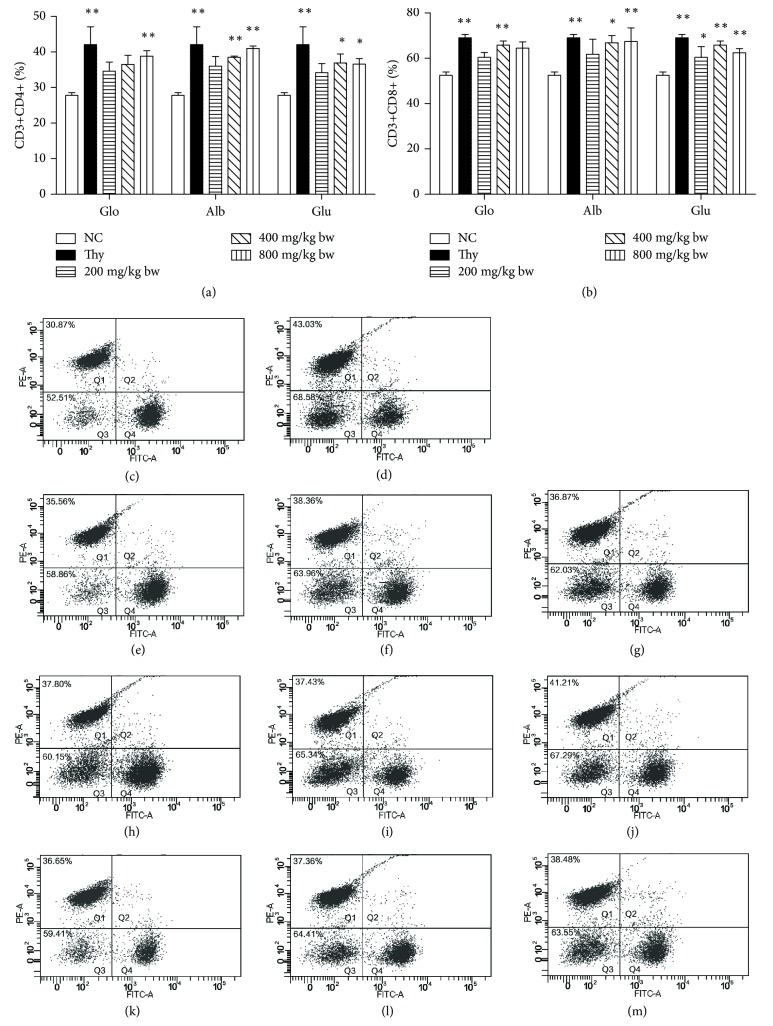
Effects of the oral administration of walnut proteins on T-lymphocyte subpopulations. Splenocytes were separated from protein-treated mice after oral administration at varying doses. Approximately 1 × 10^7^ splenocytes were used to analyze the percentages of CD3^+^/CD4^+^ (a) and CD3^+^/CD8^+^ (b) cells by flow cytometry. The control group was orally given distilled water and the positive group was given thymopeptide during the experimental period. (c) normal control group; (d) positive group; (e) Glo + L group; (f) Glo + M group; (g) Glo + H group; (h) Alb + L group; (i) Alb + M group; (j) Alb + H group; (k) Glo + L group; (l) Glu + M group; (m) Glu + H group. Values are expressed as mean ± SD. Asterisks represent statistically significant differences; ^*∗*^*P* < 0.05; ^*∗∗*^*P* < 0.001 compared with the control group.

Flow cytometry and statistical results are shown in Figure 4. Walnut proteins influenced T-cell subsets of mouse spleen. Apparent differences were observed between globin, albumin, and glutelin groups and the control group. The proportion of CD4^+^ cells increased in a dose-dependent manner in all groups except for Glu + H group. CD4^+^ cell population increased to 41% in the high-dose albumin group, a value close to that observed for the positive control group, and was significantly higher than that in the control group (*P* < 0.01).

Except for Glo + L, Glo + H, and Alb + L groups, all groups showed a significant increase in the number of CD8^+^ T lymphocytes as compared with the control group (*P* < 0.05). The percentage of CD8^+^ T lymphocytes in the moderate- and high-dose glutelin groups, moderate-dose globin group, and high-dose albumin group reached significant levels (*P* < 0.01).

### 3.6. Effect of Walnut Proteins on IgA and sIgA Levels

**Figure 5:**
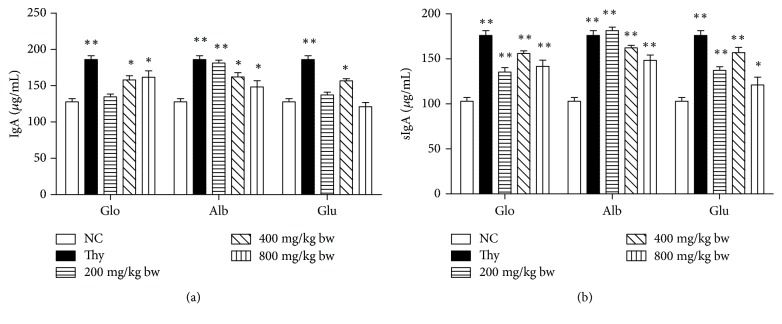
Effects of walnut proteins on immunoglobulin A (IgA) and secretory IgA (sIgA) levels in the mouse intestine. The distilled water-treated group was used as the negative control and thymopeptide was used as the positive control. The levels of IgA and sIgA in the intestine were determined by ELISA after the oral administration of the walnut proteins at varying doses. Values are expressed as mean ± SD. Asterisks represent statistically significant differences; ^*∗*^*P* < 0.05; ^*∗∗*^*P* < 0.01 compared with the control group.

In comparison with the control group, all albumin groups, moderate- and high-dose globin groups, and moderate-dose glutelin group showed a significant increase in IgA levels (*P* < 0.05; Figure 5). The increase in IgA level in the low-dose albumin group reached significant levels (*P* < 0.01). In comparison with the control group, all groups treated with three proteins showed a significant increase in sIgA levels (*P* < 0.05 or *P* < 0.01). In particular, sIgA level in the high-dose albumin group was higher than that in the positive control group.

### 3.7. mRNA and Cytokine Levels

**Figure 6:**
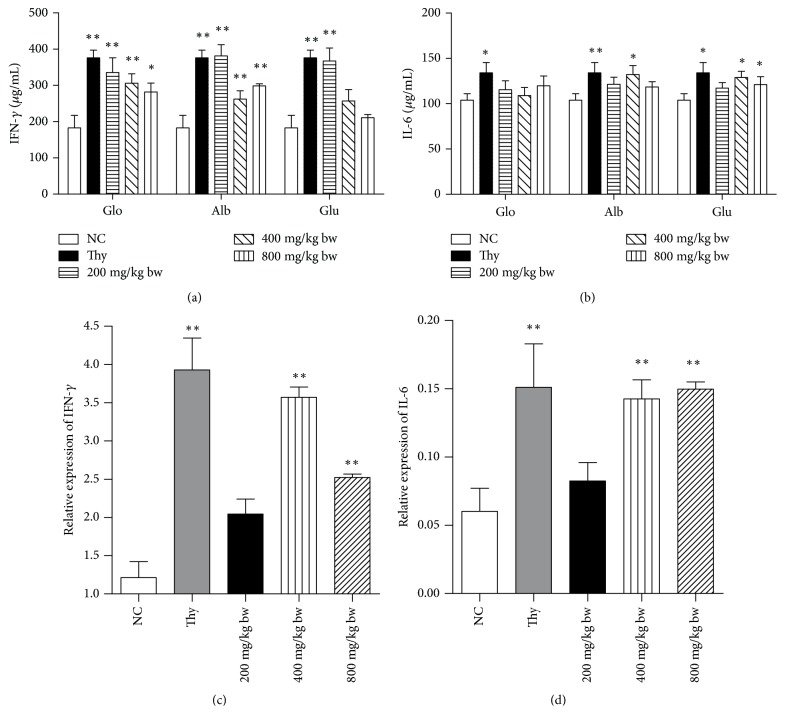
Effects of the oral administration of walnut proteins on the expression of IFN-*γ* (a) and IL-6 (b) and effects of the oral administration of albumin on mRNA expression of IFN-*γ* (c) and IL-6 (d). The control group was orally given distilled water and the positive group was given thymopeptide during the experimental period. The levels of IL-6 and IFN-*γ* were determined by ELISA after the oral administration of the walnut proteins at varying doses. Values are expressed as mean ± SD. Asterisks represent statistically significant differences; ^*∗*^*P* < 0.05; ^*∗∗*^*P* < 0.01 compared with the control group.

To evaluate the effects of walnut proteins on peritoneal immune functions, IFN-*γ* and IL-6 levels in the mice were examined (Figure 6). In comparison with the control group, those treated with different doses of three proteins showed a significant increase in IFN-*γ* levels, except for Glu + M and Glu + H groups (*P* < 0.05). The most significant effects on IFN-*γ* level were observed with low doses of three proteins (*P* < 0.01) (Figure 6(a)). In comparison with the control group, moderate-dose albumin group and moderate- and high-dose glutelin groups showed a significant increase in IL-6 level (*P* < 0.05); however, globin had no effect on IL-6 level at all tested doses (*P* > 0.05) (Figure 6(b)). After 35 days of feeding, albumin, glutelin, and globin proteins increased IL-6 and IFN-*γ* expression at different levels.

The effect of albumin on the mRNA expression of IFN-*γ* and IL6 was further examined by qPCR. In comparison with the control group, albumin groups showed a significant increase in IFN-*γ* mRNA level (*P* < 0.05) (Figure 6(c)). Furthermore, the moderate- and high-dose albumin treatment significantly affected IL-6 mRNA level as compared with control treatment (*P* < 0.01) (Figure 6(d)). High IL-6 and IFN-*γ* mRNA levels may attenuate inflammation. The moderate dose of dietary proteins upregulated the mRNA levels of anti-inflammatory cytokines IL-6 and IFN-*γ* as compared with low- or high doses of albumin.

## 4. Discussion

**Figure 7:**
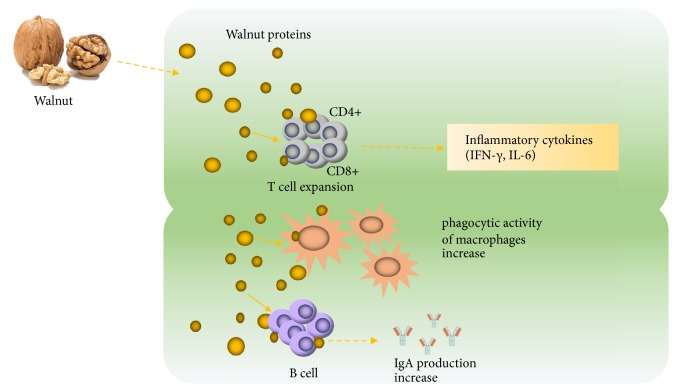
The flow chart of the mechanism of action of hydrolyzate proteins of walnut.

Walnut, a nutrient-rich food, is known to exhibit high contents of fats, proteins, vitamins, and minerals and widely planted worldwide [17–19]. In recent years, efforts have been directed to study the walnut residue that serves as a rich source of nutritional proteins. Previous studies have analyzed the composition and characteristics of walnut proteins and found these proteins exhibit antitumor and antioxidant effects [17, 19]. However, few studies have been taken to investigate the immunomodulatory effects of walnut proteins, especially the Changbai Mountain walnut. This study systematically examined the effect of three types of walnut hydrolyzate proteins—albumin, glutelin, and globin—on the immune system of mice and found they could boost mouse immune system and exhibit immunoregulatory activities (Figure 7).

The immune system is primarily composed of organs such as the spleen and thymus and cells such as lymphocytes. The change in the organ index is one of the important indicators of immune strength [20]. We determined the capability of dietary supplementation with walnut proteins to modulate the immune response based on its effects on the regulation of thymus and spleen, largest primary lymphoid organs responsible for T-cell maturation. Both thymus and spleen indices may reflect the immune function of an organism. A previous study has shown that the homogeneous polysaccharide JRP1 extracted from the epicarp of the immature fruit of* J. mandshurica *Maxim. significantly enhanced serum immune cytokine levels and spleen and thymus indices in S180 tumor-bearing mice [21]. In the present study, we found that walnut proteins markedly increased spleen and thymus indices of mice (*P* < 0.05 or *P* < 0.01). These results suggest that walnut proteins perform their immunomodulatory functions by influencing splenic enlargement.

Macrophages are important for tissue repair and host defense mechanisms as well as in morphogenetic remodeling during inflammatory responses [22]. In addition, previous studies have shown that bioactive peptides may adjust the immune response by enhancing the proliferation of lymphocytes and phagocytic capacity of macrophages [23, 24]. Our results showed that walnut proteins significantly increased the phagocytic activity of macrophages and proliferation of lymphocytes, suggesting that certain concentrations of walnut proteins may stimulate the transformation of lymphocytes into lymphoblasts. Splenic lymphocytes are immunoreactive cells and their proliferation is directly associated with cellular immunity and differentiation and proliferation of lymphocyte precursors. T-lymphocyte-mediated immune response is a critical immune response for defense against infection and T-helper (TH) cells induce B lymphocytes to produce antibodies [25]. Walnut proteins in the mouse digestive tract may be broken down into small bioactive peptides that may act as mitogens and influence lymphocyte proliferation to promote DNA replication and protein expression. This observation may be an indicative of a multienzyme system that is involved in the synthesis and secretion and may directly provide nutrition and energy, thereby increasing T-lymphocyte proliferation and improving the immune function of T cells to enhance the immunity of the animal.

CD4^+^ (T-accessory cell) and CD8^+^ (T-killer cell) are two essential types of T lymphocytes whose number and ratio are essential for maintaining stable immune response [26]. Previous studies have shown that the number of CD4^+^ cells and the ratio of CD4^+^ to CD8^+^ cells increased by hydrolytic peptides from hartshorn, wheat, hazelnut, and salmon [15, 27]. The three types of walnut proteins we studied may have similarly increased the proportion of CD4^+^ and CD8^+^ cells; however, the most significant effect was observed with albumin and glutelin. Naturally arising CD4^+^ regulatory T cells contribute to the maintenance of immune self-tolerance and control a variety of immune responses. In our study, the percentage of CD4^+^ cells significantly increased in a dose-dependent manner except in the group given a high dose of glutelin. These findings suggest that moderate and high doses of walnut proteins are suitable for reconstituting the damaged immune system through the upregulation of T-lymphocyte subsets.

Intestinal sIgA is the main effector in the mucosal barrier of the digestive tract and forms a protective layer on the mucosal surface and prevents the adhesion, colonization, and propagation of bacteria, viruses, and other pathogens on the intestinal mucosa surface. In addition, sIgA may neutralize toxins to prevent intestinal infection. Ren et al. demonstrated the hazelnut hydrolysed peptides supplementation could increase the sIgA content [15]. Gastrointestinal neuropeptide and bovine casein phosphopeptides also exerted similar effects [28]. Our results suggested that walnut proteins could improve intestinal mucosal sIgA content, therefore regulating the intestinal mucosal immunity, which were in accordance with those previously reported.

T-helper lymphocytes may be subdivided into TH1 and TH2 cells. TH1 cells produce tumor necrosis factor-alpha (TNF-*α*), IL-2, and IFN-*γ*, while TH2 cells express IL-4, IL-5, IL-6, and IL-10 [29]. IL-6 and other cytokines may interact to perform a series of immune functions [30]. IFN-*γ* belongs to the TH1 type of cytokines comprising secretions from TH1-type CD4+ T lymphocytes that may activate various immune cells and induce the production of T lymphocytes and natural killer cells, thereby influencing their proliferation as well as differentiation and antibody secretion. Type 1 immunity is characterized by production of proinflammatory cytokines, such as IFNs, TNF-*α*, IL-12, and IL-6 [31]. Our study showed that walnut proteins may increase levels of serum immune molecules at both mRNA and protein levels. Mice immunity was improved by walnut proteins through increased levels of immune cytokines, thereby maintaining the balance between Th1/Th2 immune response. This observation is similar to the results of a previous study, wherein Ndiaye et al. found that pea protein hydrolyzate inhibited the secretion of proinflammatory cytokines (TNF-*α* and IL-6) and production of activated macrophages [32]. In addition, lupine protein hydrolyzate was shown to decrease the expression of proinflammatory cytokines (TNF-*α*, IL-6, and IL-1*β*) in THP-1 macrophages [33].

## 5. Conclusions

Our results suggest that walnut protein hydrolyzates exert potential immunomodulatory effects in a dose-dependent manner. In general, walnut proteins at different doses may improve most immunological indices, and moderate doses of walnut proteins may exert sustainable immunomodulatory effects. This research contributes to an in-depth understanding of the immunomodulatory effects of walnut proteins.

## Figures and Tables

**Table 1:** The primer sequences for real-time PCR.

Gene	Primer (5′-3′)
IL-6	
Forward	ACCTGTCTATACCACTTC
Reverse	GCATCATCGTTGTTCATA
IFN-*γ*	
Forward	AGAACCATACAGCCTATTG
Reverse	TCACACTTGTCACATTCA
GAPDH	
Forward	AGGTCGGTGTGAACGGATTTG
Reverse	GGGGTCGTTGATGGCAACA

**Table 2:** Effects of walnut proteins on spleen indices in mice (*n* = 9).

Group	Globin	Albumin	Glutelin
Control	3.967 ± 0.196	3.967 ± 0.196	3.967 ± 0.196
Positive	4.951 ± 0.288	4.951 ± 0.288	4.951 ± 0.288
Low dose group	4.268 ± 0.275	4.754 ± 0.282^*∗∗*^	4.522 ± 0.204^*∗*^
Moderate dose group	4.031 ± 0.246	4.893 ± 0.260^*∗∗*^	4.585 ± 0.202^*∗*^
High dose group	4.656 ± 0.220^*∗*^	4.837 ± 0.218^*∗∗*^	5.020 ± 0.263^*∗∗*^

Comparisons among groups were analyzed by one-way ANOVA, followed by Turkey-Kramer post hoc test. *∗* indicated *P* < 0.05, and *∗∗* indicated *P* < 0.01 compared with control group.

**Table 3:** Effects of walnut proteins on thymus indices in mice (*n* = 9).

Group	Globin	Albumin	Glutelin
Control	1.257 ± 0.266	1.257 ± 0.266	1.257 ± 0.266
Positive	2.142 ± 0.234	2.142 ± 0.234	2.142 ± 0.234
Low dose group	1.672 ± 0.286	2.065 ± 0.235^*∗*^	2.005 ± 0.218^*∗*^
Moderate dose group	2.031 ± 0.249^*∗*^	2.237 ± 0.284^*∗∗*^	2.064 ± 0.240^*∗*^
High dose group	1.951 ± 0.293^*∗*^	2.094 ± 0.295^*∗*^	1.967 ± 0.203^*∗*^

Comparisons among groups were analyzed by one-way ANOVA, followed by Bonferroni's test. *∗* indicated *P* < 0.05, and *∗∗* indicated *P* < 0.01 compared with control group.
